# Neonatologist-performed point-of-care abdominal ultrasound: What have we learned so far?

**DOI:** 10.3389/fped.2023.1173311

**Published:** 2023-04-28

**Authors:** Archana Priyadarshi, Sheryl Rogerson, Rommel Cruzado, Amanda Crow, Murray Hinder, Himanshu Popat, Soundappan S. V. Soundappan, Nadia Badawi, Mark Tracy

**Affiliations:** ^1^ Westmead Hospital Neonatal Intensive Care Unit, Sydney, NSW, Australia; ^2^ Grace Centre for Newborn Intensive Care at The Children`s Hospital Westmead, Sydney, NSW, Australia; ^3^ The University of Sydney, Sydney, NSW, Australia; ^4^ The Royal Women's Hospital Neonatal Intensive Care Unit, Melbourne, VIC, Australia; ^5^ Department of Radiology, The Children's Hospital Westmead, NSW, Australia; ^6^ Department of Surgery, The Children's Hospital Westmead, NSW, Australia

**Keywords:** bowel, ultrasound, neonates, NEC=necrotizing enterocolitis, vovulus

## Abstract

This review describes the sonographic appearances of the neonatal bowel in Necrotising enterocolitis. It compares these findings to those seen in midgut-Volvulus, obstructive intestinal conditions such as milk-curd obstruction, and slow gut motility in preterm infants on continuous positive airway pressure (CPAP)-CPAP belly syndrome. Point-of-care bowel ultrasound is also helpful in ruling out severe and active intestinal conditions, reassuring clinicians when the diagnosis is unclear in a non-specific clinical presentation where NEC cannot be excluded. As NEC is a severe disease, it is often over-diagnosed, mainly due to a lack of reliable biomarkers and clinical presentation similar to sepsis in neonates. Thus, the assessment of the bowel in real-time would allow clinicians to determine the timing of re-initiation of feeds and would also be reassuring based on specific typical bowel characteristics visualised on the ultrasound.

## Introduction

A plain abdominal radiograph is the current standard investigation for detecting diseased neonatal bowel states (1). Abnormal bowel gas patterns and changes in gas distribution pattern on serial imaging during the disease process guides clinical management. Thus, multiple x-rays are often required; both AP view and left lateral decubitus positions are preferred as any free intraperitoneal gas will be contrasted by the liver (2). A plain abdominal radiograph is highly sensitive in detecting free air within the abdominal cavity confirming bowel perforation—the only universally agreed-upon indication for surgical intervention as a form of treatment (1, 3). It is unclear whether any future technology would prove to be superior to the plain abdominal radiograph in the diagnosis of diseased bowel state in neonates. However, a plain abdominal radiograph can be non-specific in many conditions such as malrotation, Hirschsprung`s, and even early necrotizing enterocolitis (NEC) (4). Further, paucity of bowel gas on the x-ray is particularly challenging to interpret clinically.

There is a growing interest in the utility of neonatologist-performed point-of-care abdominal ultrasound to assess the bowel, mainly when the plain radiograph findings are non-specific and the clinical suspicion for severe intestinal conditions is high (5). For neonatal clinicians, in the proper clinical setting, a tense tender abdomen, raised inflammatory markers, thrombocytopenia, and a plain abdominal radiograph demonstrating pneumatosis intestinalis are sufficient clinical parameters to diagnose NEC reliably. Further clinical management depends on complications such as perforation and the need for surgical exploration due to the failure of medical treatment (6). Although in well-established NEC, plain abdominal radiograph has good positive predictive value; it lacks specificity, particularly, during the early part of the disease (7). Also, with advancements in perinatal care managing more premature infants, pneumatosis intestinalis and Portal venous gas may not be evident as these infants may not be on substantial enteral feeds for the anaerobic metabolism to create intramural gas associated with intestinal wall mucosal injury (5). Thus, the utility of a plain abdominal radiograph to diagnose early NEC and the optimal timing of surgery (before the perforation occurs) are grounds to evaluate other radiological diagnostic tools such as point-of-care abdominal ultrasound to complement the findings noted on the plain abdominal radiograph and assess other features of this disease process not possible on a 2-dimensional simple abdominal film, for example, vascularity (8).

Recently, international evidence-based guidelines on point-of-care ultrasound (POCUS) for critically ill neonates and children issued by the POCUS Working Group of the European Society of Paediatric and Neonatal Intensive Care (ESPNIC) stated that the point-of-care abdominal ultrasound is helpful to detect signs of NEC; however, a pediatric radiologist should perform a detailed assessment for a definitive diagnosis (9). There is no doubt that bowel ultrasound has a steep learning curve for point-of-care neonatologists to be able to use this skill reliably (10). The availability of high-frequency linear ultrasound transducer and optimal ultrasound machine pre-sets for image acquisition are paramount (8). Repeated ultrasound scanning practice, practical strategies to manage abdominal gas artifacts, and understanding the sonographic bowel patterns in diseased states will improve diagnostic accuracy.

This review describes the sonographic appearances of the neonatal bowel in NEC. It compares these findings to those seen in midgut-Volvulus, obstructive intestinal conditions such as milk-curd obstruction, and slow gut motility in preterm infants on continuous positive airway pressure (CPAP)—CPAP belly syndrome. Point-of-care bowel ultrasound is also helpful in ruling out severe and active intestinal conditions, reassuring clinicians when the diagnosis is unclear in a non-specific clinical presentation where NEC cannot be excluded. As NEC is a severe disease, it is often overdiagnosed, mainly due to a lack of reliable biomarkers and clinical presentation similar to sepsis in neonates (11). Thus, the assessment of the bowel in real time would allow clinicians to determine the timing for the re-initiation of feeds and would also be reassuring based on specific typical bowel characteristics visualized on the ultrasound.

## A systematic approach to performing a sonographic assessment of the neonatal bowel

**Figure 1:**
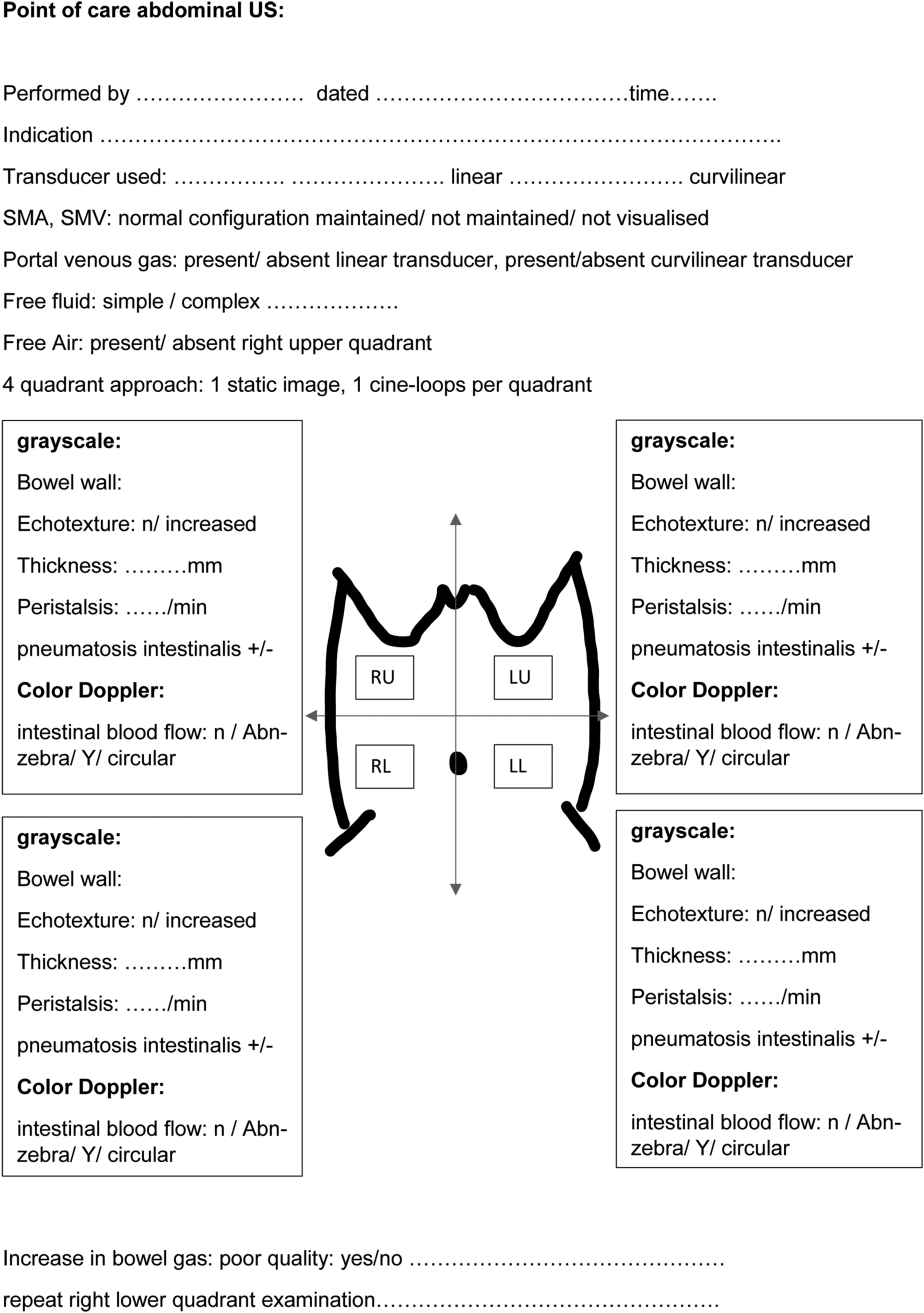
A schematic approach to perform a structured point-of-care abdominal ultrasound for bowel assessment in neonates.

The superficial location of the bowel within the abdomen warrants high-frequency linear transducers for optimal image acquisition (8). A high-frequency linear transducer, preferably more than 12 MHz, is required to obtain reasonable ultrasound images of the neonatal bowel. Linear probes with a frequency greater than 50 MHz are available, significantly improving image acquisition. In the absence of portal venous gas detected using a linear transducer, a second assessment using a curvilinear transducer, preferably 8 MHz or more, is recommended (10). This allows for greater depth penetration. A generous amount of ultrasound interface gel warmed to body temperature (by placing sterile sachets in warm water) is essential to prevent hypothermia, particularly in extremely preterm infants. Liberal use of the normothermic gel interface would promote reducing any excessive pressure application that may occur inadvertently on a potentially tender abdomen during scanning. It is essential to follow a structured approach to assess the various quadrants sequentially. A suggested way to begin is scanning from the right upper quadrant and moving sequentially across the right and left hemiabdomen (5, 8, 10) (Figure 1). In extremely preterm infants, it may not be possible to place a linear transducer separately across the upper and lower halves of the hemiabdomen. The areas of clinical interest should be re-imaged such as the right lower quadrant, which corresponds to the ileocecal region, the site most involved in NEC pathology (10). It is vital to assess the hepatic echotexture in detail as the presence of echogenic dots within the porto-hepatic vasculature is a clue to mucosal injury or breakdown of the mucosal wall secondary to perforation (5).

## Point-of-care bowel ultrasound findings in a normal bowel

**Figure 2:**
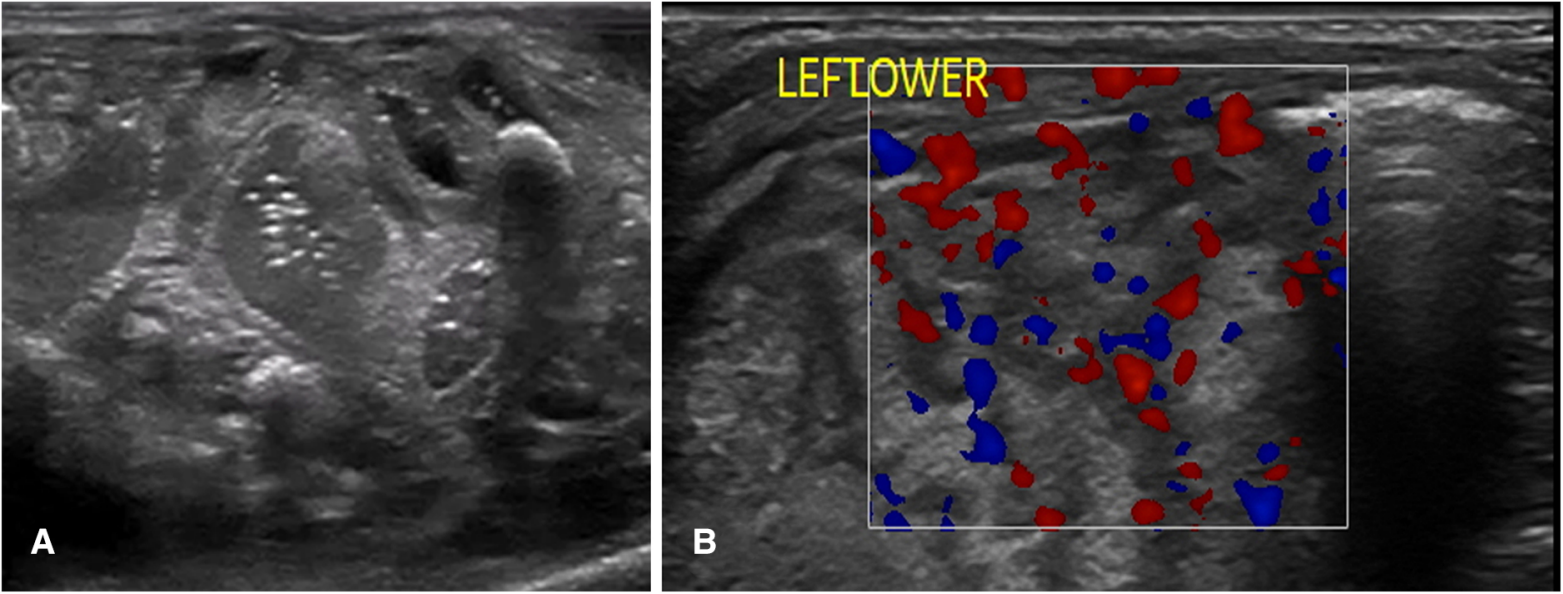
The sonographic appearance of normal bowel. (**A**) 2-D Grayscale image of normal bowel with normal intraluminal air seen as echogenic dots. (**B**) Color Doppler imaging of bowel showing normal bowel vascularity.

A normal bowel sonographic appearance is characterized by the presence of the “gut signature” forming a hypoechoic layer (Muscularis Propria) between two hyperechoic layers (Serosa and Intima) (8). The echogenic dots within the center of the lumen represent intraluminal gas, which is mobile with the position of the transducer and is a normal finding in a healthy bowel (Figure 2A). Normal bowel peristalsis appears in a worm-like motion when the bowel segment is intervened in a horizontal plain or a floral opening when seen in a cross-sectional view. This characteristic opening and closing of the bowel lumen “swirling movement” displacing the echogenic dots within the lumen that is seen in real-time represent normal bowel peristalsis that is readily appreciated despite gas artifacts and is a surrogate marker of a healthy gut. This is often the most helpful sign when there is a significant gaseous distention with the use of non-invasive respiratory support such as continuous positive airway pressure (CPAP) in extremely preterm infants (12). A neonatal bowel demonstrating more than 10 peristaltic movements over 1 min in each of the quadrants is normal and is a surrogate marker of a viable small bowel (8). BUS has the added advantage of assessing bowel wall vascularity (Figure 2B), with valuable information that can be linked to the inflammatory bowel states, particularly distinguishing severe acquired inflammatory conditions from congenital obstructive intestinal lesions, which is a distinct advantage over the plain abdominal radiograph. Faingold et al. assessed bowel wall perfusion (BWP) by counting the number of dots on the color Doppler (CD) signal/cm square and forming standardized squares on areas of interest on the bowel images using a four-quadrant approach. Normal BWP was classified as one to nine CD signal dots detected per square cm (8, 13). Bowel wall perfusion is considered present when these CD signals are reproducible or confirmed on pulsed Doppler waveforms. BWP is interpreted to be absent when no CD signal is detected at the lowest possible pulse repetition frequency without aliasing and the highest Doppler gain settings without flash artifacts. The slowest possible velocity on the color Doppler reported in the study by Faingold et al. was 0.029 m/s (13). Interpreting an absent color Doppler signal, which is suggestive of an avascular bowel wall, is prone to observer error. Thus, reducing the lowest possible velocity on the color Doppler is crucial to confirm this finding.

## Point-of-care ultrasound findings in necrotizing enterocolitis

**Figure 3:**
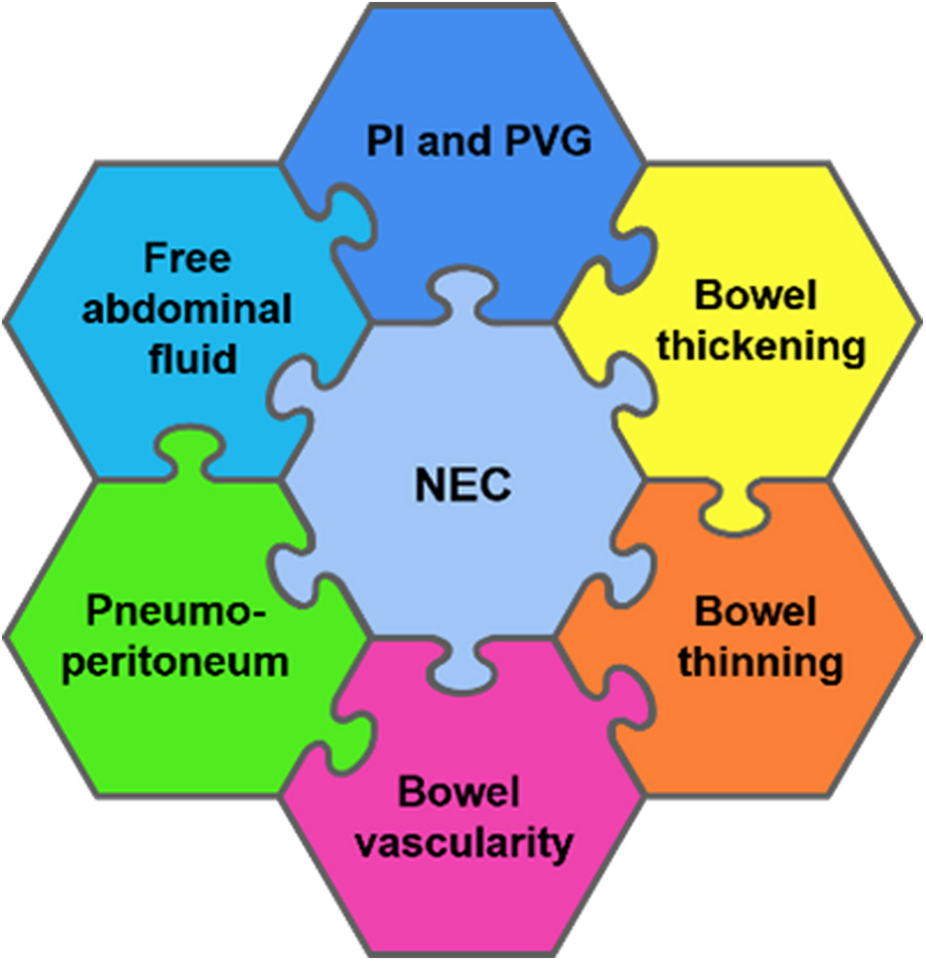
A schematic diagram showing a detailed radiological assessment of the neonatal bowel for the diagnosis of necrotizing enterocolitis (NEC). PI, pneumatosis intestinalis and PVG, portal venous gas.

The diagnostic bowel ultrasound findings in NEC have been well established by radiologists more than a decade ago, with a growing interest in this area amongst neonatologists (2). For a complete assessment of NEC and to accurately guide clinical management, the pathognomonic signs of pneumatosis intestinalis and portal venous gas are detectable on a plain abdominal radiograph; in addition, it requires an assessment for intra-abdominal fluid collection, bowel wall thickening and thinning, and vascularity, as well as guiding clinical management decisions such as continuing medical treatment or a need for surgical exploration (2, 6, 8). Micro-perforations may not be evident on a plain abdominal radiograph, and bowel wall thinning is usually a late sign. The bowel wall thinning progression to intestinal perforation secondary to severe NEC is a serious life-threatening complication (2). Thus, early detection is crucial. The BUS features of NEC include (2, 8, 14, 15) (Figure 3):

1. Bowel wall thickening or thinning
2. The loss of bowel peristalsis
3. The presence of pneumatosis intestinalis
4. The presence of portal venous gas
5. Specific patterns of vascularity in NEC
6. Pneumoperitoneum: a sign of perforation
7. Intraluminal and extra-luminal fluid collections

## Advantages of performing a point-of-care abdominal ultrasound (bowel ultrasound) as an adjunct to a plain abdominal X-ray

1. Using bowel ultrasound when the plain abdominal x-ray findings are non-specific in the clinical setting of early NEC to allow the complete radiological assessment for NEC
2. Detecting clinical deterioration inconsistent with plain abdominal x-ray abnormality changes
3. Detecting portal venous gas on point-of-care BUS compared to the plain abdominal radiograph early
4. Detecting free abdominal fluid (anechoic or echogenic) on ultrasound only
5. Detecting specific vascularity associated with NEC (in the absence of pneumatosis intestinalis) detected on ultrasound only
6. Deciding on restarting feeds post-diagnosis of NEC, using peristalsis, bowel wall assessment—normal “gut signature”, and vascularity as a guide

**Figure 4:**
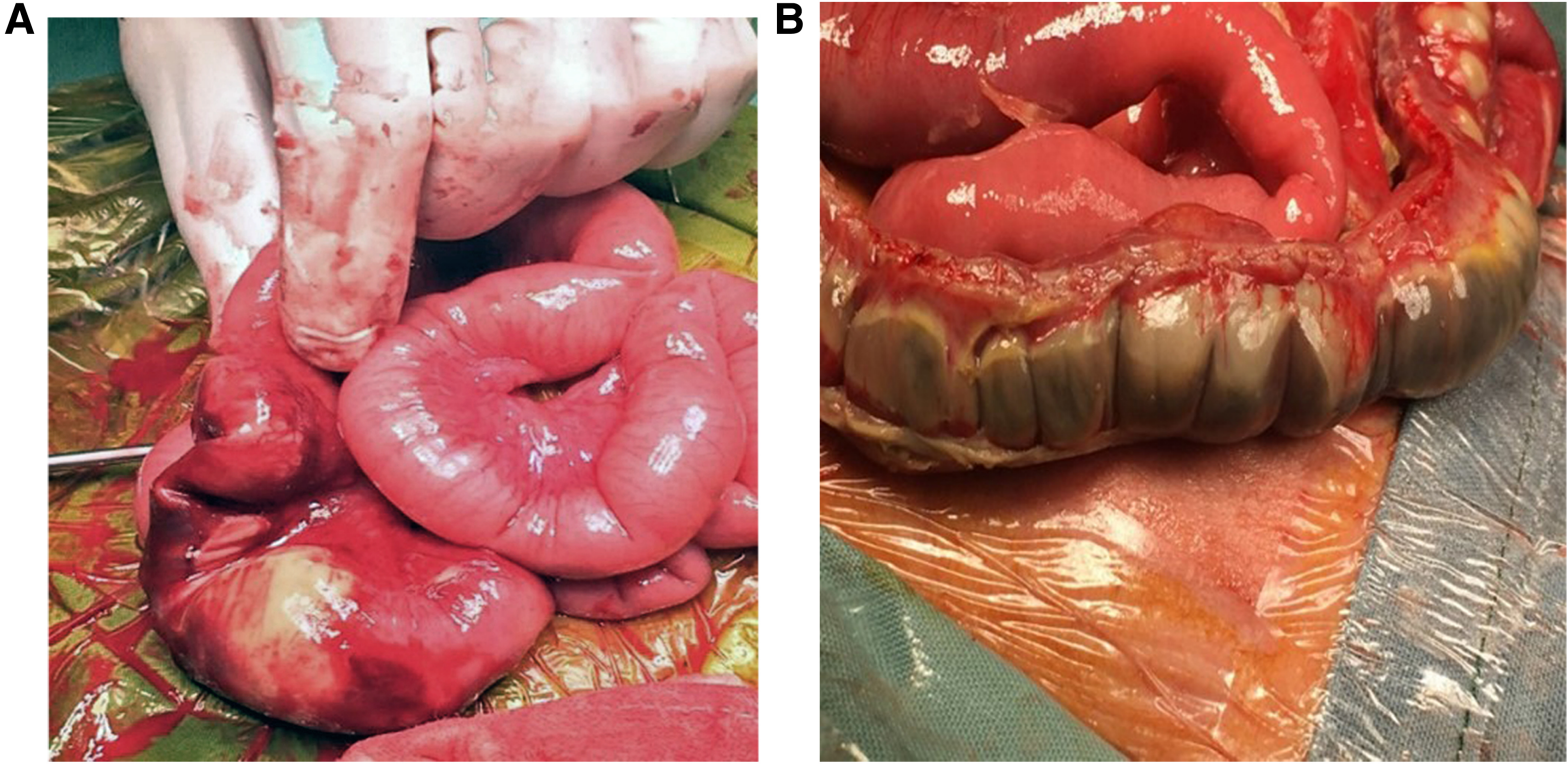
Stages of necrotizing enterocolitis (NEC). Laparotomy findings of (**A**) Localized NEC. (**B**) Severe extensive NEC with micro-perforations.

**Figure 5:**
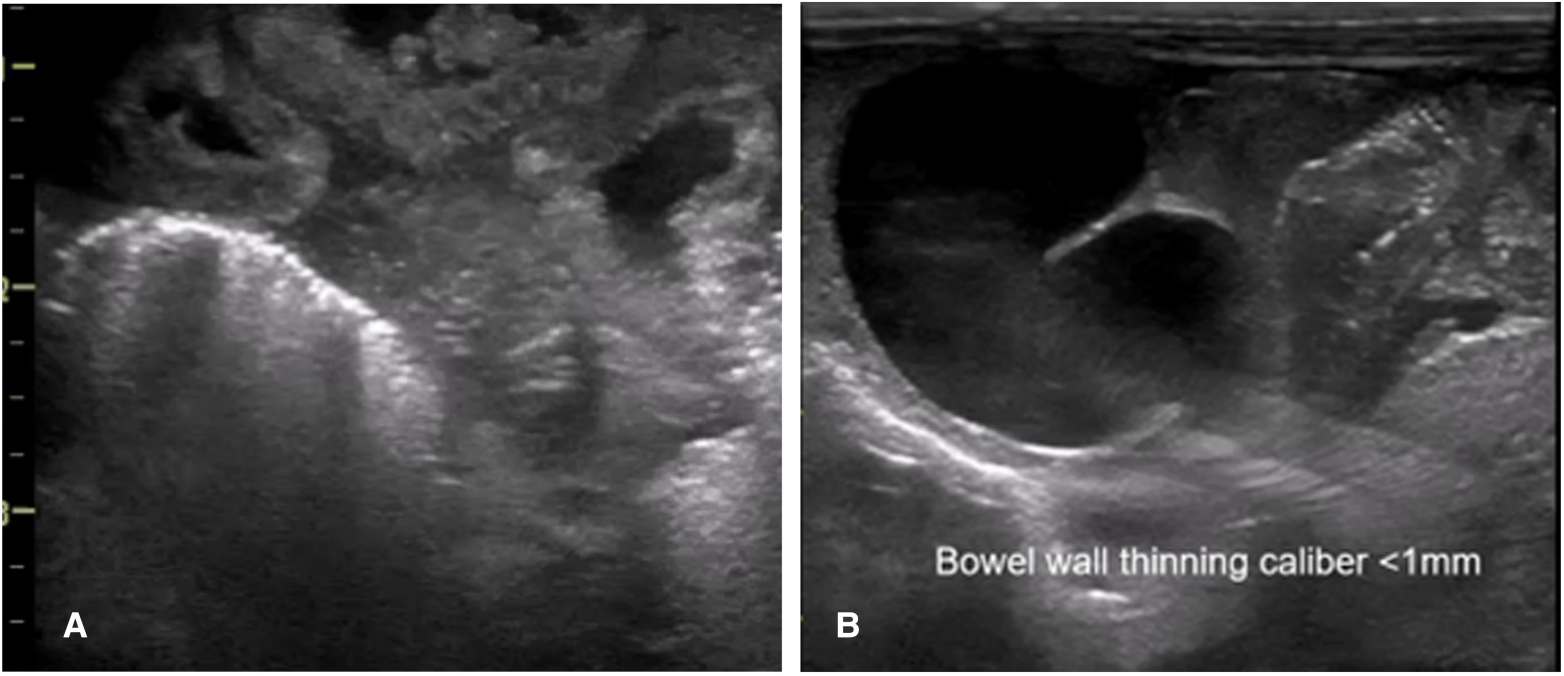
The sonographic appearance of the bowel wall during various stages of necrotizing enterocolitis on point-of-care abdominal ultrasound. (**A**) Bowel wall thickening. (**B**) Bowel wall thinning.

**Figure 6:**
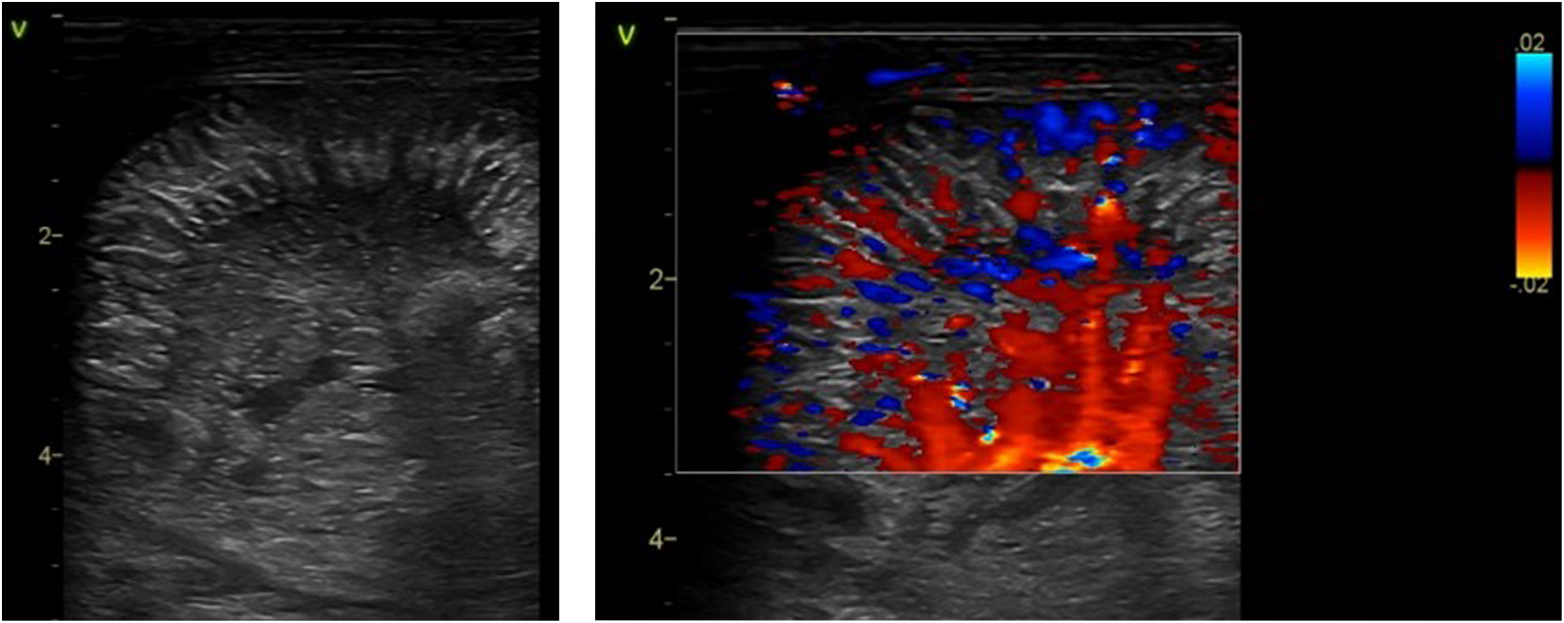
The sonographic appearance of edema and thickening of small intestinal mucosal folds as seen in necrotizing enterocolitis on point-of-care abdominal ultrasound. Grayscale imaging showing the echogenic stripping of the small intestinal folds due to edema of the valve conniventes. Color Doppler imaging of these folds showing hyperemia.

Bowel wall thickening and thinning are changes within the bowel wall seen during various stages of NEC (2) (Figure 4). Different bowel segments go through the stages of NEC from initial bowel wall thickening, which is a sign of mucosal edema and hemorrhage associated with increased vascularity with progression, to reduced perfusion, which is a thinning of the bowel wall from sloughing, and potentially bowel perforation in the severe phase of the disease (8) (Figure 5). Bowel perforation is the universally agreed indication for surgical intervention (3). However, intervention at the stage of bowel wall thinning offers clinical benefits in preventing intestinal perforation with the potentially life-threatening complication of shock and systemic inflammatory response. The small intestine (particularly the jejunum) is differentiated from the large intestine (colon) by the haustrations known as the “valve conniventes” characteristic of the small intestinal wall. The edematous small bowel shows that the thickening of these valve conniventes appears as echogenic lines on the grayscale imaging; this bowel edema represents mucosal fold edema and is not specific to NEC (2) (Figure 6). Edematous bowel segments can also be seen in obstructive and other inflammatory vascular bowel conditions such as volvulus (8).

**Figure 7:**
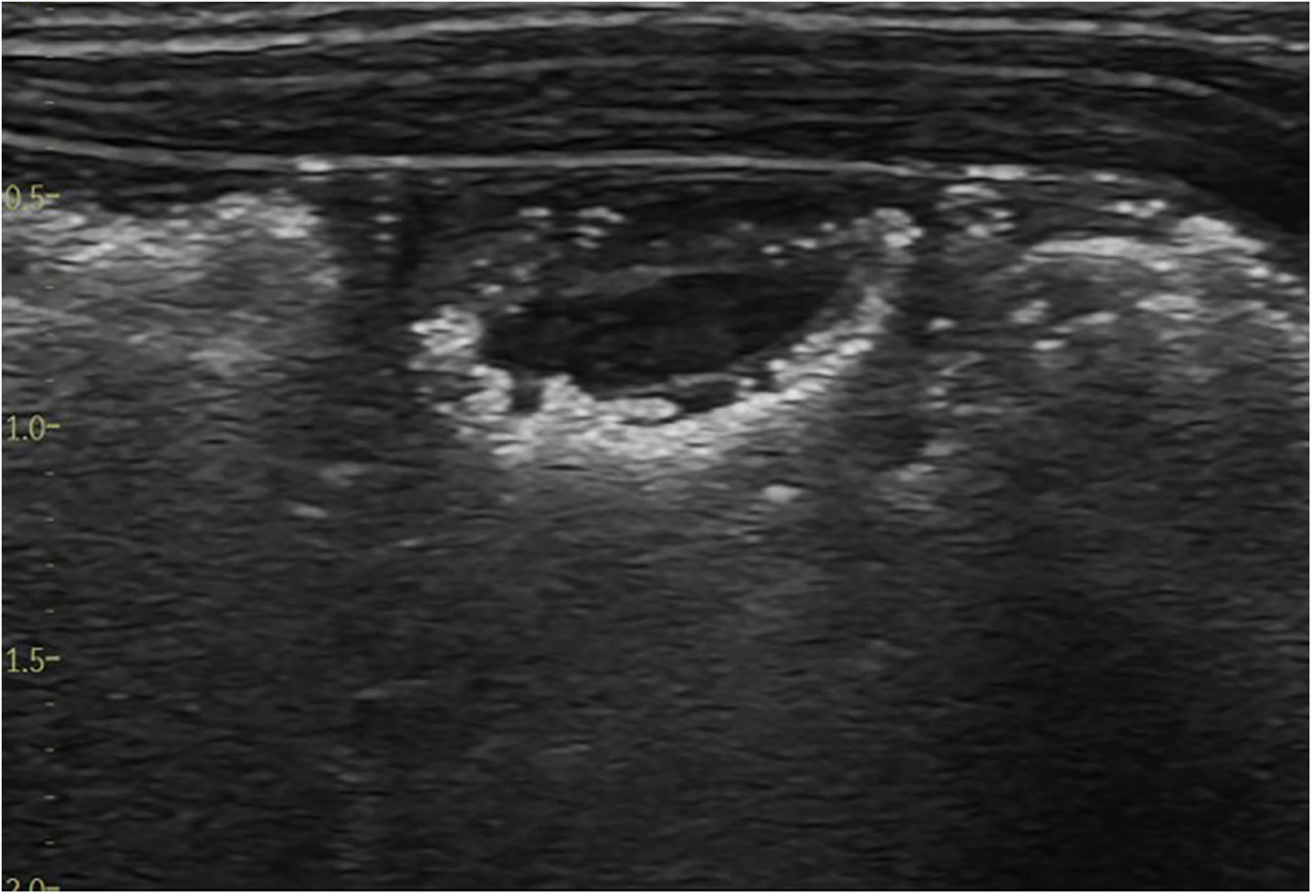
The sonographic appearance of intramural gas trapping—pneumatosis intestinalis, which is seen as white echogenic dots.

**Figure 8:**
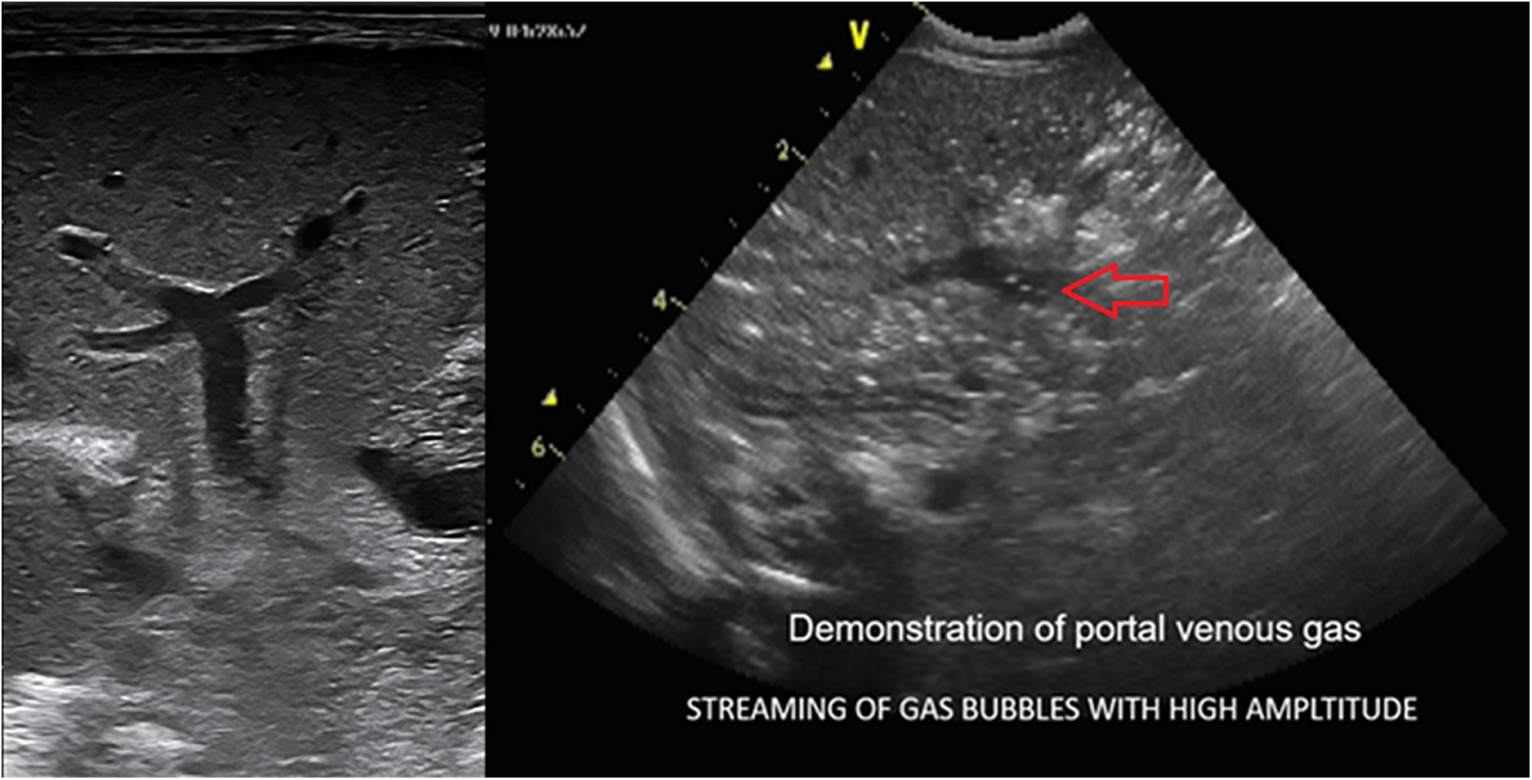
The sonographic appearance demonstrating real-time visualization of portal venous gas release seen as bright echogenic dots (streaming of gas bubbles through the portal vein). The entrapment of these bubbles within the porto-hepatic venous architecture results in abnormal hepatic echotexture seen as interspersed bright echogenic dots as opposed to the normal homogenous hepatic echotexture of a normal Liver.

The pathognomonic sign of NEC is the detection of pneumatosis intestinalis (Figure 7) or portal venous gas (Figure 8) in the appropriate clinical setting (2). These two distinct pathophysiological processes are sequelae to mucosal injury that occurs in NEC-related inflammatory edema and hemorrhage of the bowel wall. Loss of peristalsis resulting in stasis and secondary anaerobic fermentation of the intestinal contents leads to entrapment of these microbubbles within the injured bowel wall (transmural), which are seen as bright echogenic dots within the bowel wall and identified as pneumatosis intestinalis on ultrasound (8). These entrapped microbubbles-echogenic dots remain static and do not move in position with movement from the transducer (8).

Furthermore, these microbubbles escape through the venules surrounding the bowel wall into the portal vein, racing with high amplitude. The most impressive feature of BUS finding in NEC is the real-time detection of these gas bubbles spurting through the portal vein seen amidst the slow-moving, low-pressure flow of the portal venous blood. The episodic loud crackling sound effect can be heard on the application of the Doppler sound setting, with the cursor gate within the lumen of the portal vein.

As this intraluminal air traverses through the portal vein vasculature, its entrapment into the smaller tributaries of the portal venous architecture within the liver parenchyma produces a characteristic starry sky appearance of the hepatic echotexture. Bright echogenic dots scattered throughout the liver with loss of the normal homogenous sonographic liver echotexture are seen (2, 16, 17). Portal venous gas, however, is a transient phenomenon. The presence of portal venous gas is limited to 6–12 h after the disease process, and the timing of performing the ultrasound for its detection is critically time sensitive (5) should this opportunity be lost; the abnormal echotexture of the liver is indirect evidence of the recent-past process of the released portal venous gas, which can be helpful to detect NEC. Slow-emerging echogenic dots can also be seen in real-time in small bowel perforation when a slow release of these few bright echogenic dots may be visualized in the porta-hepatis. Thus, a thorough assessment of the portal vein and hepatic echotexture is extremely useful and easy to perform.

**Figure 9:**
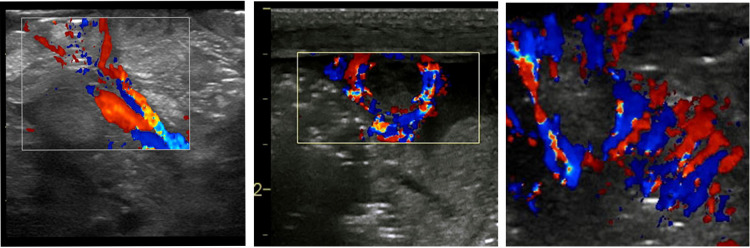
The sonographic appearance on the color Doppler showing the specific vascular patterns seen in necrotizing enterocolitis: the Y, ring, and the Zebra pattern.

Specific vascular patterns are detected in NEC (Figure 9). These characteristic vascularity patterns occur due to increased vascularity through the entire circumference of the bowel wall called the “Ring pattern”, increased vascularity through the distal-mesenteric, subserosal arteries called the “Y-pattern”, and increased flow through the mucosal folds (valvulae conniventes) called the “Zebra pattern” (10, 13). These vascular patterns require the application of the color Doppler settings, the lowest possible pulse repetition rate, and the highest Doppler gain settings without producing blooming artifacts (8).

**Figure 10:**
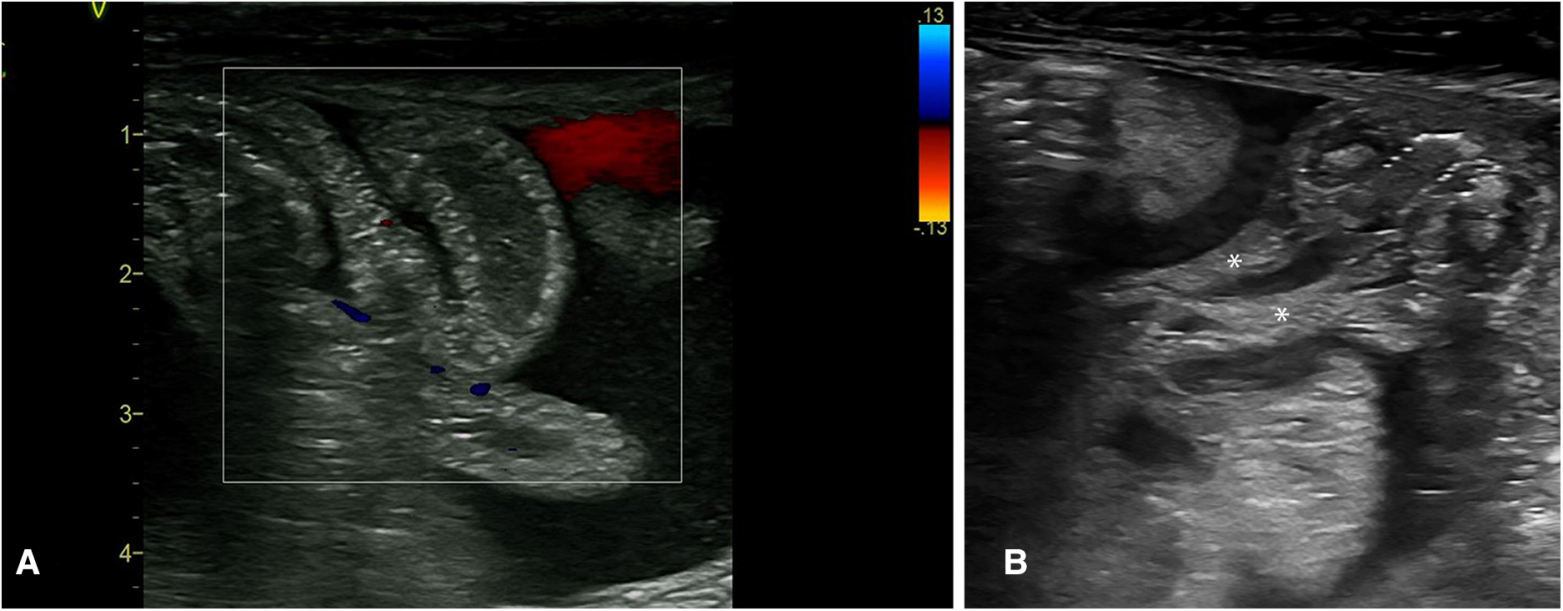
The sonographic appearance of the intra-abdominal complex fluid collections seen in necrotizing enterocolitis. (**A**) Thickened bowel loops floating within the fluid with echogenic foci (complex ascites). (**B**) Thickening of the omentum as seen within this complex fluid. * Thickened omentum.

NEC is an inflammatory condition; thus, extra-vascular fluid loss is an expected finding seen as an anechoic fluid collection. However, when this develops, a granular, echogenic appearance suggestive of exudative fluid collection is seen, suggesting a more severe stage of NEC (6). This complex fluid collection is often seen with septate partitions and floating thickened omental tissue (they appear like the stalk attached to the thickened bowel segments with loss of the normal “gut signature” of the bowel wall (Figure 10). These findings of complex ascites (floating septae and echogenic fluid collection) have a strong prediction for the need for surgical intervention. There are reports that these complex ascites (not detected on plain abdominal radiograph or clinically) were all detected in stage 3 NEC (3, 18).

## Point-of-care bowel ultrasound findings in midgut-volvulus

**Figure 11:**
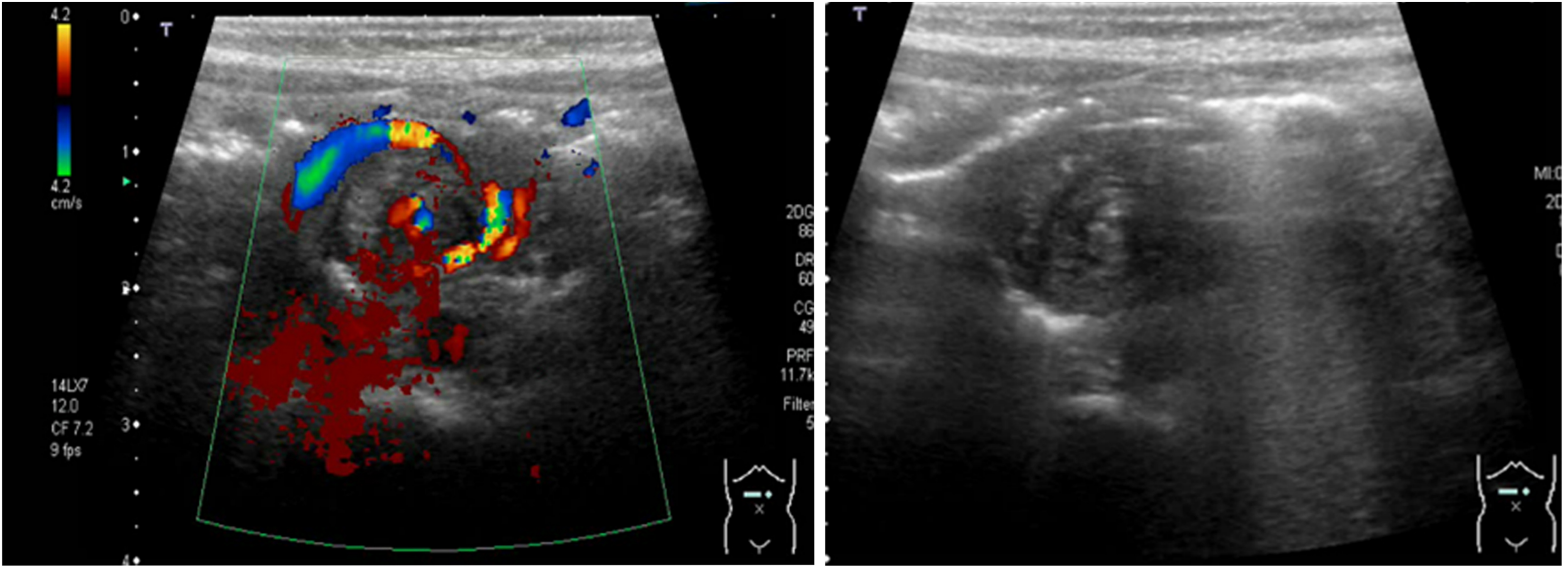
The sonographic appearance of neonatal midgut volvulus—the whirlpool sign. Color Doppler. Grayscale imaging.

Midgut-volvulus is a time-critical emergency; early detection is critical to salvage the ischemic bowel and prevent the consequences of short-gut syndrome. The detection of the “whirlpool sign” (Figure 11) is the only highly sensitive and specific sign on point-of-care abdominal ultrasound that can confirm the diagnosis of midgut-volvulus, guiding the decision for immediate surgical exploration and forms part of the clinical management algorithm in neonates with suspected volvulus (19, 20).

The primary diagnostic challenge for neonatal clinicians is to differentiate volvulus from severe NEC clinically as the line of management differs in determining the clinical decision for an urgent surgical exploration. The “whirlpool sign” is not detected in classical segmental volvulus.

Differentiating NEC from volvulus is the key clinical question. While the Whirlpool sign is pathognomonic of Mid-gut Volvulus, NEC on BUS is associated with the detection of Pneumotosis intestinalis, Portal venous gas, bowel wall thickening affecting variable non-peristaltic bowel segments, specific vascularity, loss of the gut signature, omental thickening, complex fluid collections. A scoring assessment to determine a minimum-threshold score for the diagnostic features of NEC on BUS will improve the clinical accuracy of NEC detection and timely differentiation from volvulus.

## Point-of-care bowel ultrasound findings in malrotation

**Figure 12:**
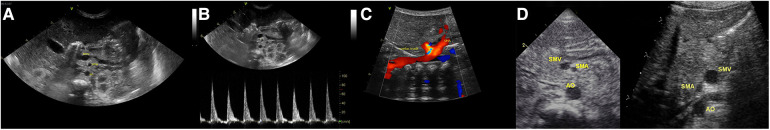
The sonographic appearance of superior mesenteric vessels—superior mesenteric artery (SMA) and the superior mesenteric vein (SMV) anatomical configuration. (**A**) Normal SMV and SMA relationship (SMV seen at 11 O’clock position to the SMA). (**B**) Arterial Doppler through the SMA confirming its anatomical location. (**C**) Sagittal image showing the relationship of the superior mesenteric artery to the aorta and the coeliac trunk. (**D**) Comparison of the normal (SMV seen at 11 O’clock position to the SMA) and abnormal positions (SMV seen at 01 O’clock position to the SMA).

This is a sagittal image showing the relationship of the superior mesenteric artery to the aorta and the coeliac trunk. Normally, the superior mesenteric vein is anterior and to the right of the superior mesenteric artery at the 11o᾽clock position. A reversal of this relationship, with the SMV positioned to the left of the SMA, suggests midgut malrotation (Figure 12). However, some patients with malrotation have a normal position of the artery and the vein. Although not diagnostic, the abnormal anatomical relationship of the SMA to that of the SMV can be a useful sign in suspecting intestinal malrotation, particularly, in preterm infants presenting with bilious aspirates. The inversion in SMA/SMV relationship demonstrated a 100% sensitivity in detecting neonatal intestinal malrotation based on the no SMV at the 11o᾽clock position to the SMA; however, normal SMA/SMV relationship can be seen in up to 29% of patients with surgically proven malrotation (19).

## Point-of-care bowel ultrasound findings in milk-curd obstruction

**Figure 13:**
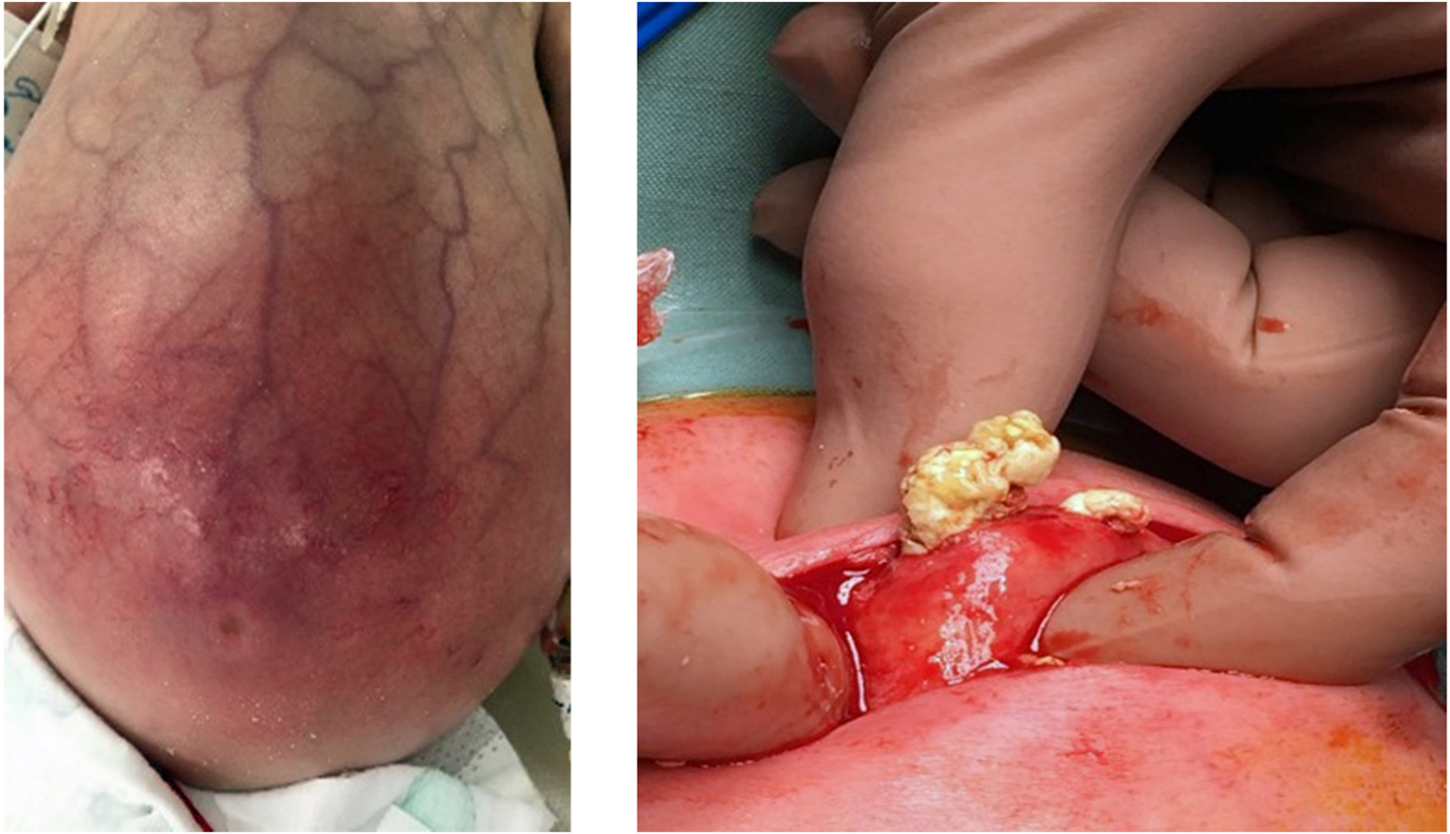
Images showing the clinical presentation of the abdomen in milk curd obstruction and the intra-operative findings in the same patient.

Milk-curd obstruction or “inspissated milk syndrome” is a rare phenomenon due to the formation of faecolith within the intestinal lumen, typically affecting preterm infants (21). With the change in feeding practices and the increased survival of extremely preterm infants, this condition has evolved from term formula-fed infants to human-milk-fed preterm infants on fortifiers, with a surge in sporadic cases presenting as acute intestinal obstruction (22). While the cause of faecolith formation remains unknown, disproportionate absorption of additive nutrients—calcium and fatty acids—to water within an immature dysmotile intestinal segment of the premature gut has been described (22–24). The dramatic presentation of abdominal distention, feed intolerance, bilious vomiting, and constipation, which are all consistent with intestinal obstruction in the clinical setting of a preterm infant on non-invasive respiratory support and fortified milk feeds, is a diagnostic challenge to the neonatologist and can be suspected as NEC (Figure 13). Several reported cases in preterm infants have been managed with a presumptive diagnosis of NEC, pre-operatively, only to find milk-curd obstruction confirmed upon diagnostic laparotomy.

The classic radiological sign of milk-curd obstruction on a plain abdominal radiograph includes scattered heterogeneous opaque masses often described as white or grey, which are either homogenous or with a “soap bubble̕” or “ground glass̕” appearance pattern and surrounded by a black halo representing air (25). Unlike the meconium plug syndrome where due to mucus the plug sticks to the intestinal mucosa, faecolith in milk-curd does not stick to the mucosal surface due to its fatty-acid calcium stone, which escapes around this rigid mass, producing the characteristic halo (25). Although plain abdominal radiograph findings in classical milk curd are typical, further diagnostic evaluation to substantiate the diagnosis of milk-curd obstruction is required as it is often considered the diagnosis of exclusion. A contrast study enema has been performed in a few reported cases (24). The most common site of milk-curd formation is the terminal ileum; thus, a contrast enema study is not beneficial. Colonic milk-curd inspissation has been described (26), and depending on the degree of obstruction, evacuation of stools may be possible; however, the potential risk of pressure-induced perforation, peritonitis, and septicemia makes it a less preferred option.

The utility of neonatologists-performed point-of-care abdominal ultrasound findings in milk-curd obstruction has never been described. Based on the clinical and laboratory parameters of plain abdominal radiograph findings and utilizing point-of-care BUS, we may be able to scale down the clinical possibility of NEC, adding value to the diagnostic assessment and interpretation of cases with milk-curd obstruction. Early diagnosis of milk-curd obstruction may prompt the cessation of milk-feed fortifiers and prevent complications such as bowel perforation.

**Figure 14:**
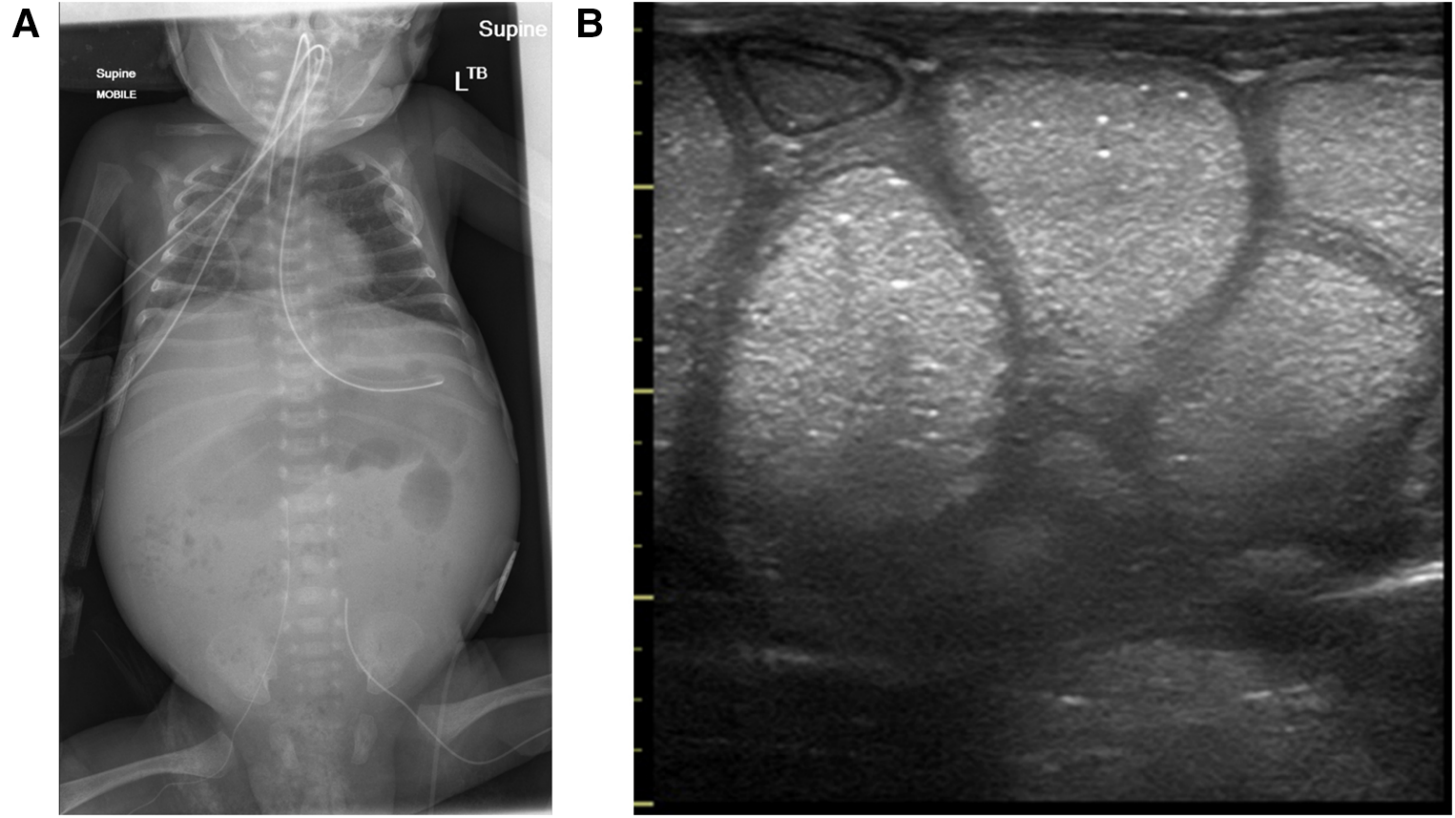
Milk-curd obstruction concurrent x-ray and point-of-care abdominal ultrasound findings. (**A**) Plain abdominal x-ray showing paucity of bowel gas with a soap-bubbly appearance on the right. (**B**) The sonographic appearance of the bowel showing islets of echogenic homogenous material within the lumen maintaining the normal bowel-wall sonographic appearance.

Milk-curd obstruction on BUS appears as islets of homogenous bright echogenicities within the intraluminal cavity, clearly demarcating the bowel wall, maintaining the “gut signature”, differentiating it from NEC (Figure 14; Table 1).

**Table 1:** Comparison of point-of-care abdominal ultrasound findings in necrotizing enterocolitis and milk-curd obstruction.

Ultrasound finding	Necrotizing enterocolitis	Milk-curd obstruction
Pathognomonic findings	Portal venous gas, increased echogenicity of the liver parenchyma, Pneumatosis Intestinalis-intramural gas (within the bowel wall)	Bowel lumen filled with echogenic homogenous opacities
Bowel wall	“Gut signature” is lost	“Gut signature” is maintained
Free fluid	Anechoic fluid—simple ascites is usually associated with early NEC; micro-perforations result in complex ascites suggestive of severe NEC	Not a feature unless there is perforation
Color Doppler: Intestinal-blood flow	Increased vascularity: Zebra, Y vascularity, a circular sign may be detected.	No increased vascularity

## Point-of-care bowel ultrasound findings in CPAP-belly syndrome

The sonographic characteristics of “CPAP-belly syndrome” include the presence of a normal “gut signature” of the bowel wall, normal bowel vascularity, normal bowel peristalsis, and intraluminal bowel gas seen as echogenic dots that move with peristalsis. NEC is a severe intestinal condition, and with the lack of biomarkers, it is often overdiagnosed. Thus, screening neonates with BUS is useful in reassuring clinicians, preventing overdiagnosis of NEC and the implications of empiric antibiotics, and cessation of enteral feeds.

## Conclusion

Thus, in conclusion, the utility of point-of-care abdominal ultrasound in the hands of point-of-care neonatologists can enhance the diagnostic accuracy of intestinal conditions in newborn infants along with using standard plain abdominal radiographs as the first line of investigation. BUS allows further comprehensive bowel screening vital to make the correct diagnosis. There is no doubt that bowel ultrasound has a steep learning curve. Still, practice and clinicopathological correlations, particularly intra-operative findings, have resulted in meaningful interpretations of the bowel ultrasound findings to allow us to learn this skill to guide clinical management for newborn infants with suspected diseased bowel conditions.
